# Hospitals and Medical Schools

**Published:** 1905-04-22

**Authors:** 


### HOSPITALS AND MEDICAL SCHOOLS.

The following circular letter has been addressed by King Edward's Hospital Fund for London to the secretaries of the 12 London hospitals with medical schools:—

"81 Cheapside, E.C., April 12th, 1905.

"The hon. secretaries beg to call your attention to the following resolution, which was passed by the Executive Committee of this Fund on March 10th last, and approved:—

"'That the Executive Committee, having considered the recommendation of Sir Edward Fry's Committee (par. 27), advises the general council to notify to the hospitals that no grants will be made from the King's Fund as from and after January 1st, 1906, to any hospitals which make payments to, or on account of, their medical schools out of general funds subscribed for the relief of the sick poor.'

"The Executive Committee think it desirable to offer some explanatory remarks on the subject, which may be of assistance to the committees of the hospitals concerned.

"Where the hospital and school form a single institution, or where subscribers have been in the habit of subscribing to the hospital funds, knowing that grants are made from those funds to the school, it will be necessary in future to keep separate accounts for the school, both in respect of receipts and expenditure. All money given to the hospital on and after January 1st, 1906, not specially earmarked by the donor, should be paid direct into the general account, and nothing should be paid out of the general account to, or on account of, the medical school. It follows that the medical school should be treated as a separate establishment, and that a sufficient payment for rent, repairs, insurance, and all value received from the hospital by the medical school should be made by the school to the hospital.

"In regard to interest on capital given to the hospitals for the joint purposes of hospital and school, an apportionment may be necessary, and the Executive Committee will be glad to receive and consider any scheme of apportionment which, if and when agreed to, would finally dispose of that part of the question as far as it relates to this Fund.

"These observations apply only to hospitals which desire to obtain grants from this Fund, and the conditions laid down are merely those which are precedent to grants being made."

